# Acute hepatitis A infection‐associated hemophagocytic lymphohistiocytosis in adult presenting as impending acute liver failure: A case report and literature review

**DOI:** 10.1002/ccr3.5334

**Published:** 2022-02-07

**Authors:** Panotpol Termsinsuk, Piyaporn Sirisanthiti

**Affiliations:** ^1^ Gastroenterology unit School of Medicine Institute of Medicine Suranaree University of Technology Nakhon Ratchasima Thailand; ^2^ Division of Hematology Department of Internal Medicine Maharat Nakhon Ratchasima hospital Nakhon Ratchasima Thailand

**Keywords:** case report, hemophagocytic lymphohistiocytosis, hemophagocytic syndrome, hepatitis A, hepatitis A virus, infection‐associated hemophagocytic syndrome

## Abstract

Hemophagocytic lymphohistiocytosis has been reported as a severe complication of various viral infections but unusual for the hepatitis A virus. We report a case of 25‐year‐old man with hepatitis A infection‐associated hemophagocytic lymphohistiocytosis and impending acute liver failure to emphasize the importance of early diagnosis and treatment of this condition.

## Graphical abstract

Diagnosis of acute HAV‐associated HLH is challenging because of its rarity. Clinical, laboratory, and hemophagocytic evidence are crucial for early diagnosis even with incomplete diagnostic criteria. Standard treatment for this condition is not well‐define. However, early immunosuppressive therapy is a cornerstone for improving survival and evading fatal complications.

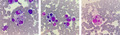

## 1 INTRODUCTION

Hemophagocytic lymphohistiocytosis (HLH) is a rare, life‐threatening condition characterized by clinical and laboratory evidence of extreme systemic inflammation resulting from uncontrolled immune activation.^1^ Diagnosis of HLH required any five of eight criteria based on the HLH‐2004 guidelines, which consist of fever, splenomegaly, at least two lineages of cytopenia, hypertriglyceridemia and/or hypofibrinogenemia, elevated ferritin, low or absent NK cell activity, elevated soluble CD25, and hemophagocytosis in bone marrow, spleen, or lymph nodes.^2^ HLH was categorized into familial and acquired. Acquired HLH usually arises in association with malignancy, infection, and autoimmune disease. Among infectious etiology, virus is the major contributor including the Epstein–Barr virus (EBV), cytomegalovirus (CMV), and human herpesvirus (HHV).^3^, ^4^ Nevertheless, HLH has been rarely reported as the consequence of acute hepatitis A infection. Delayed diagnosis and treatment in unrecognized cases has resulted in high mortality.^5^ Herein, we present a case of 25‐year‐old man with acute high‐grade fever, hepatomegaly, thrombocytopenia, and rapidly progressive hepatic dysfunction. Acute hepatitis A infection‐associated HLH with impending acute liver failure was diagnosed and treated successfully with corticosteroid and intravenous immunoglobulin.

## 2 CASE REPORT

A 25‐year‐old man presented with high‐grade fever for one day without any significant organ‐specific symptoms. His past medical history was unremarkable. He denied alcohol consumption, herbal intake, and illicit drug use. A physical examination revealed body temperature (BT) of 39.5°C, non‐tender hepatomegaly (liver span of 14 cm) without icterus, signs of chronic liver stigmata, ascites, and splenomegaly.

On admission, laboratory tests were significant for aspartate aminotransferase (AST) 303 unit/L (normal range 5–34), and alanine aminotransferase (ALT) 374 unit/L (normal range 0–55). The bilirubin level, alkaline phosphatase, complete blood count (CBC), and coagulogram were unremarkable (Table 1). The hepatitis panel was positive for hepatitis A IgM. Whereas hepatitis A IgG, hepatitis E IgM/IgG, dengue NS1 antigen, dengue IgM/IgG, EBV IgM/IgG, hepatitis B surface antigen, hepatitis B core IgM/IgG, hepatitis C antibody, anti‐nuclear antibody (ANA), and HIV antibody were negative. An abdominal ultrasonography showed hepatomegaly without space‐occupying lesions. The visualized hepatic vein, portal vein, and biliary system were also unremarkable. Therefore, acute hepatitis A infection became the diagnosis based on his clinical presentation and initial laboratory findings.

**TABLE 1:** Laboratory findings of patient in current report with acute hepatitis A infection‐associated hemophagocytic lymphohistiocytosis

Laboratory findings	Day 1	Day 3	Day 4^†^	Day 5	Day 7^‡^	Day 11	Day 30	Day 150
Hemoglobin, g/dl	16.1	14.9	15.0	16.0	13.1	13.3	13.5	14.8
Leukocytes x10^9^/L	3.7	2.8	3.6	4.3	7.9	7.7	14.4	5700
Platelet count x10^9^/L	140	109	100	110	114	273	164	276
Total bilirubin, mg/dl (normal 0–1.2)	0.7	–	3.5	4.4	7.3	2.8	1.2	0.7
Direct bilirubin, mg/dl (normal 0–0.5)	0.4	–	2.8	3.5	4.6	1.3	0.4	0.1
AST, IU/L (normal 5–34)	303	1486	5652	2872	526	74	43	25
ALT, IU/L (normal 0–55)	374	1537	5397	5794	3560	838	134	45
Alkaline phosphatase, IU/L (normal 40–150)	98	–	150	143	153	183	113	89
INR	–	1.07	1.34	1.29	1.24	1.02	–	1.01
Ferritin, ng/ml	–	–	59332	53620	16272	1635	1308	756
LDH, IU/L (normal 0–248)	–	–	6255	2541	688	154	243	252
Triglyceride, mg/dl	–	–	80	82	–	–	–	–
Fibrinogen, mg/dl	–	–	324	300	–	–	–	–

Abbreviations: AST, Aspartate aminotransferase; ALT, Alanine aminotransferase; INR, International normalized ratio; LDH, Lactate dehydrogenase.

^†^ Start high‐dose dexamethasone

^‡^ Start intravenous immunoglobulin (IVIG) 400 mg/kg/day for 5 days

During admission, he had persistent high‐grade fever (BT: 39–39.5°C) with rapid deterioration of the liver biochemistry (Table 1). Serial blood tests revealed mild thrombocytopenia along with international normalized ratio (INR) prolongation, elevated serum lactate dehydrogenase (LDH), and hyperferritinemia. Given the rapidly progressive hepatic dysfunction and severe systemic inflammation, impending acute liver failure due to acute hepatitis A infection‐associated HLH was suspected. The differential diagnosis of this condition included severe sepsis due to complicated bacterial septicemia, severe leptospirosis and rickettsial infection. Thus, hemoculture for bacteria and indirect immunofluorescent (IFA) test for Leptospira and rickettsia were sent and became negative.

**FIGURE 1:**
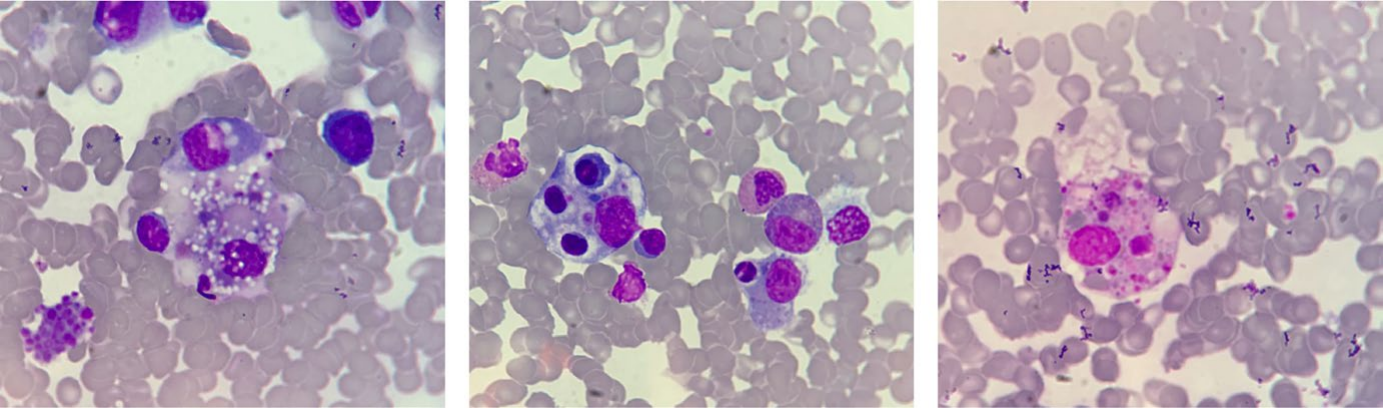
Bone marrow aspiration demonstrate multiple foci of hemophagocytosis

A bone marrow study was urgently performed to establish the diagnosis. Increased histiocytes with multiple foci of hemophagocytosis were demonstrated on aspiration smears (Figure 1). Pulse intravenous dexamethasone at a dose of 40 mg/day was immediately started resulting in defervescence and marked improvement of the liver biochemistry, inflammatory markers, and thrombocytopenia within 72 h (Table 1). The bone marrow biopsy confirmed hemophagocytosis without any histologic and immunohistochemical evidence of lymphoma and EBV. Abdominal and chest computer tomography (CT) unrevealed lymphadenopathy that could suggest lymphoma. Intravenous immunoglobulin (IVIG) at a dose of 400 mg/kg/day for five days was then administered at Day 7 of the illness with a satisfactory result. Oral dexamethasone was tapered off over the next four weeks. There was no evidence of clinical and laboratory recurrence after a five‐month follow‐up.

## 3 DISCUSSION

Hepatitis A virus (HAV), the RNA virus in the *Picornaviridae* family, is one of the most common causes of acute hepatitis worldwide.^6^ Primary transmission of HAV is the fecal‐oral route via contaminated food and water. The incidence of HAV infection tends to be higher in low socio‐economic and poor sanitation areas. Furthermore, the high‐risk population includes males who have sex with males, HIV‐infected patients, close contact of HAV‐infected cases, and illicit drug users.^7^ Non‐specific viral illness is the most common presenting symptom and usually self‐limited.^7^, ^8^ However, 0.5% of acute HAV‐infected patients were complicated with acute fulminant hepatitis causing a high mortality rate up to 80%.^8^, ^9^

In addition to acute fulminant hepatitis, hemophagocytic lymphohistiocytosis (HLH) has been reported as a rare complication of acute HAV infection. HLH is the catastrophic hyperinflammatory condition caused by overstimulation of hemophagocytic activity of the macrophage in reticuloendothelial organs. For HAV‐associated HLH, the virus triggers T‐lymphocytes to release the inflammatory cytokines and stimulate massive hemophagocytosis.^10^, ^11^ Delayed diagnosis and treatment of acute HAV infection‐associated HLH result from the rarity of the disease entity and non‐specific clinical presentation. Thus, the authors conducted a literature review from the PubMed, Google Scholar, and Cochrane databases till September 2021, using the following terms: “hepatitis A”; “hepatitis A virus”; “HAV”; “hemophagocytosis”; “haemophagocytosis”; “hemophagocytic lymphohistiocytosis”; “haemophagocytic lymphohistiocytosis”; and “HLH.” The publications were extracted only for the case report in adults (aged over 15 years at diagnosis). A total of 20 case reports (including the current patient) were reviewed, and the clinical data comprising the baseline characteristics, laboratory findings, clinical progression, treatment, and outcomes were obtained (Table 2).

**TABLE 2:** Overview of the reported cases of acute hepatitis A infection‐associated hemophagocytic lymphohistiocytosis in adult

Case	Authors	Year	Age	Sex	Presenting symptoms	Clinical progression	Treatment	Outcome	Remarks
1	McPeake JR^28^	1993	20	F	Fever, headache, vomiting	Confusion, pancytopenia, jaundice	Hydrocortisone 50 mg IV every 6 h and IVIG	Alive	Still's disease
2	Kondo H^22^	1995	49	F	Fever, jaundice	Persistent fever, rash, pancytopenia	1.5 g pulse IVMP and 250 mcg rhG‐CSF	Alive	
3	Wu CS^24^	1995	23	M	Fever, jaundice for 3 weeks	Progressive pancytopenia, and hepatosplenomegaly	IV steroid	Died	HCV carrier ALF, DIC, GIB
4	Kyoda K^29^	1998	40	M	Fever, anorexia	Self‐limited	No specific treatment	Alive	
5	Onaga M^30^	2000	19	F	Fever, malaise, nausea, vomiting	Rapidly progressive thrombocytopenia	1000 mg IVMP then tapered dose	Alive	
6	Watanabe M^11^	2002	45	M	Headache, fatigue, fever	Self‐limited	No specific treatment	Alive	
7	Watanabe M^11^	2002	41	M	Fever, hepatitis	Self‐limited	No specific treatment	Alive	HCV carrier
8	Ishii H^16^	2003	37	M	Fever, fulminant hepatitis with partial clinical improvement	Died on Day 66 of admission. Autopsy confirmed HLH.	IVMP, vincristine cyclophosphamide, plasma exchange	Died	Aspergillosis abscess
9	Tai CM^31^	2005	32	M	Fever, malaise, splenomegaly	–	IVIG	Alive	
10	Lee HJ^32^	2007	26	F	Fever, pancytopenia	–	Cyclosporine, dexamethasone, and IVIG	Alive	
11	Tuon FF^5^	2008	24	F	Nausea, vomiting, myalgia, jaundice, fever (improved)	Persistent jaundice at Day 30 after onset with anemia, fever, and hepatosplenomegaly	IVIG 400 mg/kg/day for 5 days and G‐CSF for 3 days	Alive	
12	Cho E^23^	2010	48	M	Fatigue, jaundice	Progressive jaundice with fever, rash, and acute kidney injury	IV steroid, G‐CSF	Alive	
13	Seo JY^17^	2010	22	F	Nausea, anorexia (improved)	Day 16 after onset, she developed fever, jaundice, pancytopenia, and hepatosplenomegaly	IVIG 400 mg/kg/day	Died	ALF, DIC, intraperitoneal bleeding
14	Park YH^33^	2011	24	F	Fever, anorexia	Progressive jaundice, anemia, and thrombocytopenia	Dexamethasone 10 mg/m^2^/day IV and cyclosporin 3 mg/kg IV	Alive	
15	Park HS^25^	2012	28	F	Fever, cytopenia	–	Not available data	Died	
16	Saxena P^18^	2014	15	M	Fever, anorexia, nausea, vomiting (improved)	Day 15 after onset, he developed progressive jaundice, fever, pancytopenia, and hepatosplenomegaly	Oral prednisolone (25 mg/day) then tapered‐off within 10 days	Alive	
17	Alhaddad OM^34^	2016	19	F	Jaundice and fatigue	Progressive pancytopenia	1 g IVMP for 3 days, oral prednisolone 60 mg/day	Alive	
18	Mallick B^35^	2019	21	M	Fever, jaundice, nausea, vomiting	Progressive jaundice and pancytopenia	IVIG 400 mg/kg/day for 5 days	Alive	G−6‐PD deficiency
19	Dogan A^36^	2021	50	M	Nausea, vomiting, fever, fatigue, and jaundice	–	IVIG 0.5 g/kg/day for 2 days and dexamethasone 10 mg/m^2^/day IV	Alive	
20	Our case	2021	25	M	High‐grade fever, hepatomegaly, mild thrombocytopenia	Persistent fever, rapidly progressive hepatitis, thrombocytopenia, and impending ALF	Dexamethasone 10 mg IV every 6 h (tapered‐off within 4 weeks), IVIG 400 mg/kg/day for 5 days	Alive	

Abbreviations: ALF, Acute liver failure; DIC, Disseminated intravascular coagulation; G‐6‐PD, glucose‐6‐phosphate dehydrogenase; GIB, Gastrointestinal bleeding; HCV, Hepatitis C virus; HLH, Hemophagocytic lymphohistiocytosis; IV, Intravenous; IVIG, Intravenous immunoglobulin; IVMP, Intravenous methylprednisolone; rhG‐CSF, Recombinant human granulocyte colony‐stimulating factor.

The clinical characteristics of the reported cases from the literature review were analyzed using SPSS software for Windows, version 18 (SPSS, Inc.,) and delineated in Table 3. The mean age at diagnosis was 30.5 ± 11.2 years (range from 15 to 50 years), and 12 patients (60%) were male. The most common presenting symptoms were fever (95%) and jaundice (50%). On physical examination, hepatomegaly and splenomegaly accounted for 91.6% and 81.3%, respectively. In comparison with the study of MacKinney‐Novelo et al.,^7^ the presenting symptoms among uncomplicated acute HAV infection and HAV‐associated HLH based on the reviewed data were comparable. However, hepatosplenomegaly was the predominant physical sign in HAV‐associated HLH compared to those without HLH (81.3%–91.6% vs. 7%–78%).^7^, ^12^, ^13^

**TABLE 3:** Clinical characteristics of the reported cases of acute hepatitis A infection‐associated hemophagocytic lymphohistiocytosis

Characteristics	Value	Data available
Age at diagnosis, years	30.5 ± 11.2	20/20
Male gender, *n* (%)	12 (60.0%)	20/20
Clinical manifestation		
Fever	19 (95.0%)	20/20
Jaundice	10 (50.0%)	20/20
Nausea, vomiting	7 (35%)	20/20
Anorexia	4 (20%)	20/20
Hepatomegaly	11 (91.6%)	12/20
Splenomegaly	13 (81.3%)	16/20
Hemoglobin, g/dl	11.8 ± 4.2^†^, 6.6 ± 3.5^‡^	19/20^†^, 8/20^‡^
Leukocytes x10^9^/L	3.7 (2.1–7.8)^†^, 3.0 (2.5–3.9)^‡^	19/20^†^, 8/20^‡^
Platelet x10^9^/L	85 (37–147)^†^, 80 (15–108)^‡^	19/20^†^, 11/20^‡^
At least two lineages of cytopenia	9 (45.0%)	20/20
Total bilirubin, mg/dl	7.8 (2.0–30.0)^†^, 22.0 (6.5–30.0)^‡^	19/20^†^, 13/20^‡^
AST, IU/L	1212 (351–2982)^†^, 906 (351–5652)^‡^	15/20^†^, 10/20^‡^
ALT, IU/L	731 (350–2456)^†^, 1797 (400–5794)^‡^	18/20^†^, 10/20^‡^
Alkaline phosphatase, IU/L	299.5 (162.0–411.5)^†^, 150 (119–321)^‡^	12/20^†^, 3/20^‡^
Ferritin, ng/ml	3558.3 (1499.7–59332.0)^†^, 34051 (5724–61466)^‡^	13/20^†^, 4/20^‡^
LDH, IU/L	3071 (1447–5679)^†^, 5439 (2938–6255)^‡^	14/20^†^, 6/20^‡^
Triglyceride, mg/dl	386 (138–579^†^, 493 (212–520)^‡^	11/20^†^, 5/20^‡^
Fibrinogen, mg/dl	267 (218–462^†^, 296.7 (171.2–440.5)^‡^	7/20^†^, 3/20^‡^
NK cell activity, %	7^‡^	1/20^‡^
Soluble CD25 (sIL2R), IU/ml	2590 (1920–4870)^†^	3/20^†^
Hemophagocytosis in bone marrow	13 (100.0%)	13/20
Bone marrow aspiration	13 (100.0%)	13/20
Bone marrow biopsy	10 (100.0%)	10/20
Complete five of eight diagnostic criteria^§^	7 (36.8%)	19/20
Treatment		
Steroid	12 (63.2%)	19/20
Chemotherapeutic agent	3 (15.8%)	19/20
IVIG	8 (42.1%)	19/20
Spontaneous resolution without treatment	3 (15.8%)	19/20
Mortality rate	4 (20%)	20/20

Data are presented as mean ± standard deviation or median (interquartile range) and number (proportion) of patients with a condition according to the available data.

Abbreviation: AST, Aspartate aminotransferase; ALT, Alanine aminotransferase; LDH, Lactate dehydrogenase; IVIG, Intravenous immunoglobulin; sIL2R, Soluble interleukin‐2 receptor.

^†^ Value at diagnosis.

^‡^ Value at maximal disease activity.

^§^ Based on HLH‐2004 guideline.

Acute hepatitis A infection was diagnosed by HAV IgM in all reported cases. The test usually positive at the onset of the symptoms gave a high diagnostic sensitivity and specificity of more than 99%.^14^, ^15^ Therefore, delayed diagnosis for acute uncomplicated HAV infection was not observed, and the diagnosis was usually made on their first admission of viral illness in the reviewed data. In contrast to HAV‐associated HLH, four cases were diagnosed at 15–30 days after the improvement of their first episode of acute hepatitis A infection causing a delay in treatment and high mortality.^5^, ^16^, ^17^, ^18^ The explanation for the delayed onset of HLH in these four cases was unclear. Nonetheless, it could be a result of delayed immune activation or a relapse of acute HAV infection (“relapsing hepatitis”) causing an immunologic rebound and trigger more severe systemic inflammation.

Thrombocytopenia was the initial hematologic abnormality in HAV‐associated HLH based on the reviewed data (Table 3). In childhood HAV infection, thrombocytopenia is usually caused by immune thrombocytopenia (ITP).^19^, ^20^ However, in adult HAV infection, thrombocytopenia could be the early sign of HLH and usually progress to pancytopenia at the maximal disease activity. Thus, the superimposed HLH should be considered in acute HAV‐infected patients who have thrombocytopenia on the initial presentation. Moreover, cytopenia of at least two lineages was required to complete the diagnostic criterion according to the HLH‐2004 guidelines.^2^ Nevertheless, only 45% of HAV‐associated HLH patients exhibited two lineages of cytopenia based on the reviewed data (Table 3). Thus, this criterion was ineligible to contribute to the early HLH diagnosis since more than two cytopenia usually occurred in the latter course of HLH.

Among uncomplicated HAV‐infected patients, liver biochemical changes usually manifested as a mixed hepatocellular and cholestatic pattern. The average peak of total bilirubin (TB) was 7–9 mg/dL, whereas the mean AST and ALT levels could be higher than 1000 IU/L, which were similar to those with HAV‐associated HLH based on the reviewed data.^7^, ^12^ However, the patient of the current report had rapidly progressive hepatic dysfunction characterized by the increase of the AST and ALT levels above twentyfold of the upper normal limit within 48 h along with the INR prolongation (Table 1). All of these findings postulated the impending acute liver failure and raised suspicion of superimposed HLH.

Liver injury in HLH typically occurs in the early phase as a result of a cytokines storm and could rapidly progress to acute liver failure and death.^21^ Hepatic dysfunction would lead to various biochemical alterations, including hypertriglyceridemia (due to impaired lipoprotein lipase activity), hypofibrinogenemia causing subsequent coagulopathy, disseminated intravascular coagulation (DIC), and multiorgan dysfunction.^21^ The elevation of the LDH and serum ferritin was a result of cellular injury and severe systemic inflammation. Nonetheless, hypofibrinogenemia and hypertriglyceridemia were not observed in the patient of the current report, as the HLH was diagnosed in the early course.

Bone marrow aspiration and/or biopsy usually demonstrates hemophagocytosis in most reported cases. Bedside microscopic evaluation of aspiration smears provided prompt HLH diagnosis in the patient of the current report even though there were only three compatible diagnostic criteria including fever, hyperferritinemia, and bone marrow hemophagocytosis. The presence of at least five of eight criteria for the HLH diagnosis was observed only in seven patients (36.8%) based on the reviewed data (Table 3). Thus, the combination of the clinical findings, laboratory evidence of systemic inflammation, organ damage, and bone marrow hemophagocytosis was crucial for the HLH diagnosis even though there were incomplete HLH‐2004 criteria. The HLH diagnostic criteria were usually fulfilled in the later course of the disease and could delay the diagnosis and treatment.

Standard treatment protocol for acute HAV‐associated HLH was not well‐established. Various treatment regimens for HAV‐associated HLH have been reported, including corticosteroid, intravenous immunoglobulin (IVIG), and chemotherapeutic agent either single or combination therapy (Table 2). Twelve patients (63.2%) and eight patients (42.1%) in the reviewed data were treated with corticosteroid and IVIG, respectively. A chemotherapeutic agent was prescribed in three patients (15.8%) and usually combined with corticosteroid and/or IVIG. A granulocyte colony‐stimulating factor (G‐CSF) was used as an adjunctive treatment in three reported cases.^5^, ^22^, ^23^ Nonetheless, three patients (15.8%) were spontaneously resolved without treatment.

The patient of the current report was successfully treated with intravenous high‐dose dexamethasone and IVIG without any evidence of clinical and laboratory recurrence at a 5 months follow‐up. However, three reported cases of HAV‐associated HLH died upon corticosteroid and/or IVIG treatment due to acute liver failure and superimposed infection.^16^, ^17^, ^24^ Although corticosteroid and IVIG seemed to be the effective treatment options in HAV‐associated HLH, treatment initiation in the latter disease course eventually contributed to the poor treatment outcomes, especially in those who already had significant organ failure. A chemotherapeutic agent was not initiated in the patient of the current report, as he was in clinical and laboratory remission after corticosteroid and IVIG treatment.

The overall mortality rate of HAV‐associated HLH was 20%, whereas the 30 days mortality and overall mortality of adult HLH were 20%–44% and 50%–75%, respectively.^25^, ^26^, ^27^ The cause of death in the reported cases of HAV‐associated HLH was acute liver failure and superimposed disseminated fungal infection.^16^, ^17^, ^24^ Delayed immunosuppressive therapy and presence of acute liver failure at treatment initiation might be associated with death according to the reviewed data (Table 2).

In conclusion, this case report emphasized the importance of early diagnosis and treatment of HAV‐associated HLH. Thus, clinical, laboratory, and hemophagocytic evidence is crucial for diagnosis and would encourage prompt treatment even with incomplete HLH diagnostic criteria. Although the standard treatment approach in adult HAV‐associated HLH is not well‐defined, early immunosuppressive therapy would be the cornerstone for improving survival and evading fatal complications. Hence, further clinical studies could elaborate a more effective treatment paradigm for HAV‐associated HLH.

## CONFLICT OF INTEREST

The authors declare that they have no competing interests.

## AUTHOR CONTRIBUTIONS

PT provided clinical and pathological diagnosis, performed literature review, images acquisition, and wrote the manuscript. PS acquired information of the patient, provided pathological diagnosis, supervised for the treatment and followed up. All authors have read and approved the final manuscript.

## ETHICAL APPROVAL

Not applicable.

## CONSENT

Written informed consent was obtained from the patient for publication of this case report and any accompanying images. A copy of the written consent is available for review by the Editor of this journal.

## Data Availability

Data sharing is not applicable to this article as no datasets were generated.
